# Insect applications to open wounds by chimpanzees in the wild: first insights from East African chimpanzees

**DOI:** 10.1038/s41598-025-16582-5

**Published:** 2025-08-25

**Authors:** Kayla Kolff, Daniela Acosta Flórez, Alessandra Mascaro, Simone Pika

**Affiliations:** 1 Comparative BioCognition, Institute of Cognitive Science, Osnabrück University, Osnabrück, Germany; 2 Department of Anthropology, Faculty of Arts and Sciences, Yale university, New Haven, United States of America

**Keywords:** Animal medication, Self-medication, Other-regarding care, *Pan troglodytes schweinfurthii*, Prosocial behaviour, Entomotherapy, Ecology, Evolution, Psychology, Animal behaviour

## Abstract

Medicative behaviours are widespread among animals, and chimpanzees in the wild may exhibit a newly identified form involving the application of insects to open wounds. To date, insect applications to wounds have only been reported in a single community of Central chimpanzees (*Pan troglodytes troglodytes*). Thus, we report observations of similar behaviours in Eastern chimpanzees (*P. t. schweinfurthii*) of the Ngogo chimpanzee population, in Kibale National Park, Uganda. Between November 2021 and July 2022, we observed six individuals (three males, three females) applying flying insects to their own wounds (*N* = 5) and, in one instance, to the wound of a conspecific. These observations demonstrate a generally consistent sequential pattern of insect applications in both Eastern and Central chimpanzees. Although the extent and potential medicinal function of this behaviour remain unclear, we propose three hypotheses to inform future research, focusing on insect selectivity, social transmission, and prosociality. In conclusion, the findings suggest that insect applications are more widespread than currently documented, and provide a basis for investigating their acquisition, social dynamics, and potential relevance to the evolution of human medicinal behaviours.

## Introduction

Traditional medicinal behaviours are a cultural characteristic^1^ that human societies have assembled over thousands of years^2–4^. These behaviours have been passed on through oral transmission, observation, and direct copying (i.e., social learning)^5^, with traditional medicine continuing to play a vital role in global healthcare^6,7^ (but see also^8,9^. Understanding the origins of these medicinal behaviours – such as the development or use of herbal medicines, ointments, entomotherapy (the medicinal use of insects^3^, and modern pharmaceuticals – requires examining how knowledge of medicinal compounds may have emerged, been transmitted, and refined over time.

Evidence suggests that medicinal plants and fungi were used by our extinct hominin ancestors, the Neanderthals (*Homo neanderthalensis*) as early as 50,000 years ago, with some of these plants still in use today^4,10^. For instance, German chamomile (*Matricaria chamomilla*) shown to have little nutritional value^10^, and today used for different purposes including anti-inflammatory, antispasmodic, and sedative effects, as well as the treatment of gastroprotective disturbances^11^. Similarly, entomotherapy^3,12,13^ has been used in some cultures for centuries. For instance, in China, approximately 300 insect species across 70 different genera have been identified as having medicinal properties and are included in traditional Chinese medicine^14^. Similarly, in Burkina Faso, traditional healers use insect species from six different orders for a variety of medicinal purposes^15^. The insects are used to treat a wide range of ailments, including digestive and skin disorders^3^, and their documented effects entail anti-inflammatory, antimicrobial, antiviral, immunological, analgesic, antibacterial, diuretic, anaesthetic, and antirheumatic effects^3,12^.

This raises a central question: how did humans first recognise and harness such medicinal properties, and how might this have evolved? One explanation, suggested by scholars, is that throughout human history, people have observed nonhuman animals (hereafter animals) treating their illnesses using plant secondary compounds, non-nutritional substances, or bioactive substances^16–18^. These observations have contributed to the identification of plant-based treatments with potential benefits for humans^19^. This explanation raises a further question: how did such behaviours emerge in animals themselves? A growing body of research across the animal kingdom – from invertebrates to primates^20–30^ – has shown that various animals engage in medicative behaviours. These include the use of natural substances such as plant matter^31–33^, clay^29^, or millipedes (*Sechelleptus* spp.)^34^ or for prophylactic or therapeutic purposes, often involving antiparasitic effects (see also^35^.

Most existing research has focused on cases of self-medication involving plant materials, with only limited examples of animal-derived substances^35^. Furthermore, until recently, the use of medicinal substances to treat others was thought to be rare in animals, however, emerging evidence suggests it may be more widespread than previously assumed^35–37^. These findings contribute to the ongoing debate about the existence of prosocial behaviours in nonhuman primates^38,39^, defined as actions intended to benefit another and, in humans, primarily driven by empathic concerns for others^40,41^. Hence, the social context of wound tending may represent a crucial candidate for enhancing our understanding of prosocial and therapeutic behaviours in animals and the evolution of human medicinal care.

One compelling example of such behaviour comes from recent observations on Central chimpanzees (*Pan troglodytes troglodytes*) from the Loango National Park, Gabon. These observations documented a potentially significant example of both self- and other-directed medication involving the application of insects to open wounds – not only their own but also those of related and unrelated group members^37^. Over a period of 15 months, a total of 19 self-directed events (five adult males, one adult female, one juvenile female) and three other-directed events (one female, two males; one kin-directed and two non-kin-directed events) were observed. These events followed a consistent behavioural sequence:

- A flying insect was caught.
- It was immobilised by being placed and/or squeezed between the lips.
- The insect was applied to the exposed surface of the wound with the fingers and moved across it using the fingers or lips.
- It was removed from the wound using either the mouth or fingers and discarded.

These observations expand our existing knowledge of animal medication to include allo-medication of open wounds involving the use of animal matter^37^. In addition, since documented events of insect application in chimpanzees in the wild have only recently been observed, questions arise regarding the behaviour’s uniqueness, distribution, context-specificity, and links to ecological and sociodemographic factors. Understanding these aspects helps determine whether the behaviour reflects an intrinsic response, a community-specific innovation, or part of a broader pattern, with implications for chimpanzee self-medication, social transmission, and prosocial behaviour. Here, we provide first insights into insect application in another chimpanzee subspecies – Eastern chimpanzees (*Pan troglodytes schweinfurthii*) – from the Ngogo population, Kibale National Park, Uganda. We report three events: two events of self-directed application, and one of other-directed application. These observations offer comparative insight into the distribution and significance of insect-based wound treatment in chimpanzees and its relevance to the evolution of prosocial and health-related behaviours.

## Results

In total, we observed five events involving insect-related behaviour with wounds. These events varied in terms of individual identity, sex, and age class of the applicator, wound location, and peering occurrence (Table 1). We further report on three events due to the availability of video footage and visibility, entailing two self-directed applications, and one other-directed application.

**Table 1:** Summary of reported events of insect applications based on video footage and tabulated observational data as a function of date, ID (wounded individual), sex (M = Male; F = Female), age class of the wounded individual (adolescents: 8 < x < 16; adults: x ≥ 16^42^, wound location, applicator (self-applied, *N* = 5; or applied by a conspecific, *N* = 1), presence of bystander peering during insect application, and availability of video footage. The numbers in the example events match the three events described in the results.

Date	ID	Sex	Age class	Wound location	Applicator	Peering	Video footage	Example events
**Western community**								
23/11/21	Damien	M	Adolescent	Left leg	Self	Yes	Yes	2
23/11/21	Damien	M	Adolescent	Left leg	Etta James	Yes	Yes	3
**Central Community**								
28/11/21	Clayton	M	Adolescent	Left leg	Self	No	No	
3/12/21	Abrams	M	Adult	Right lower back	Self	No	Yes	
13/12/21	Fisher	F	Adult	Left upper arm	Self	No	No	
18/03/22	Fricka	F	Adolescent	Right leg	Self	Yes	Yes	1

### *Event 1 (see Video S1)*. *An adolescent female chimpanzee*,* Fricka*,* self-applied a flying insect to an open wound on her inner right thigh*

**Fig. 1:**
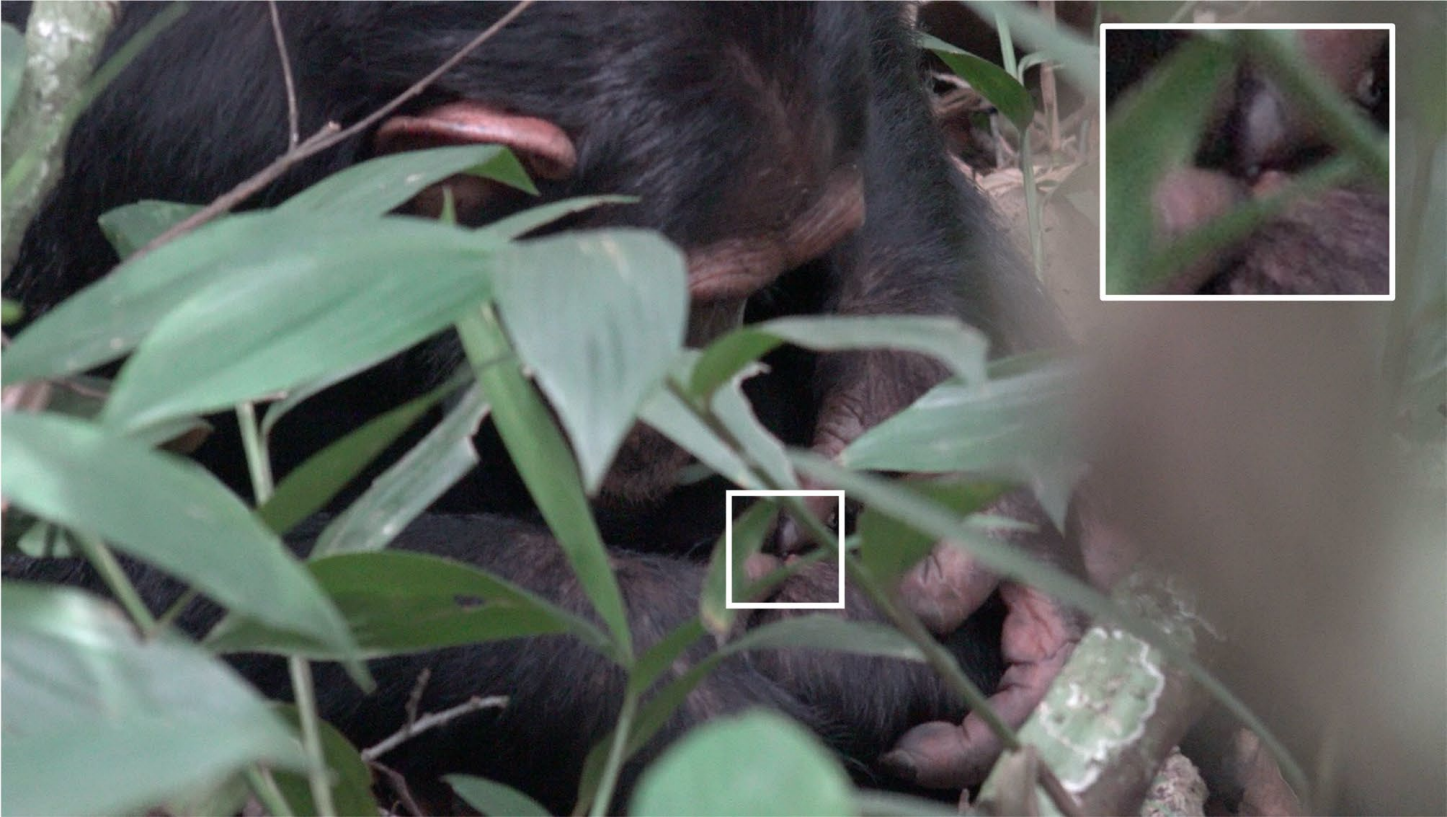
An adolescent female, Fricka, applied a flying insect to a wound on her inner right thigh. The wound is indicated by the white square, and the insect, visible as a blue-coloured object between her thumbs, is shown in the zoomed-in view within the white-bordered square in the top-right corner.

This event involved an adolescent female, Fricka, who was observed applying an immobilised insect to an open wound on her inner right thigh (approximately < 5 cm in diameter; Fig. 1; Video S1, 00:16 − 01:42). She pressed the insect (with only the abdomen visible) onto the wound surface and picked multiple instances of white residue from the insect. At one point, while grooming the wound with her fingers, the insect was pushed aside onto nearby fur, but she subsequently retrieved it and placed it back onto the wound surface (Video S1; 01:23 − 01:30). During the application, white residue was observed on seven separate occasions on her lower lip and thumb (Video S1; timestamps: 00:16, 00:33 [at which time white residue was also visible on the insect part], 00:36, 00:41, 01:04, 01:18), and white viscous residue was also observed attached to the insect part when discarded from the wound (Video S1; 01:43). Of these instances, two demonstrated clear ingestion (Video S1; 00:41, 01:18), whilst in other cases the residue was observed to adhere to her lower lip and chin area after being brought to her mouth (Video S1; 00:17 − 00:27, 00:35 − 00:36, 00:38), making it unclear whether ingestion occurred. Throughout the insect application activity, she produced orofacial sounds – teeth clacks and lip smacks^43,44^.

### *Event 2 (see Video S2). An adolescent male chimpanzee*,* Damien*,* caught and applied a flying insect to an open wound on his inner right calf*

The event involved Damien, an adolescent male, who captured a flying insect within two attempts, using a grasping movement on leaves while locomoting (Video S2; 00:04 − 00:18). Damien proceeded to press the captured and immobilised insect to his wound on his inner right calf (approximately < 5 cm in diameter) and was observed re-applying the insect on two occasions. He held the insect in his mouth or between his lips two times before reapplying (Video S2; 00:56 − 01:02, 01:06 − 01:08). At the end of the application, he placed the insect on the edge of his lower, looked at it before discarding it (Video S2; 01:44 − 01:56). An unrelated adult male, Wayne and Damien’s younger adolescent sister peered at the activity at different points; Wayne stopped peering and walked away before the end of the event (Video S2; 00:22 − 01:58) and Etta-James continued peering until Damien started to groom her (Video S2; 01:10 − 02:23).

### *Event 3 (see Video S3). An adolescent female chimpanzee*,* Etta James*,* applied a flying insect to an open wound on the inner right calf of her adolescent brother*,* Damien*

This event took place shortly after *Event 2* (approximately 90 s). An adolescent female and sister to Damien, Etta James, was observed applying an immobilised flying insect to Damien’s open wound (approximately < 5 cm in diameter). Etta James had successfully captured a flying insect by using a quick grasping movement on the nearby leaves (observational recording) before proceeding to press the insect on Damien’s wound (Video S3; 00:04 − 00:22). During the application, she pressed the insect on Damien’s wound and held between her lips into her mouth three times, once before reapplication (Video S3; 00:10 − 00:13) and twice before attempted reapplication (Video S3; 00:25 − 00:30, 00:51 − 00:54). After, the second attempted application, she walked away with the insect still in hand (Video S3; 00:55 − 01:14) rested nearby and inspected the insect before discarding it (K.K. personal observation). Their older adult maternal brother, Rollins, was resting nearby, who began peering at the activity during the first attempted reapplication until Damien repositioned himself (Video S3; 00:34 − 00:56).

## Discussion

The present study aimed to provide insight into insect application to wounds in a different chimpanzee subspecies. We observed five events and describe three in detail involving the application of a flying insect onto an open wound, resulting in two self-directed and one other-directed application by Eastern chimpanzees from the Ngogo population. The observations suggest a general sequential pattern in the application of insects to wounds: first, the insect was captured from nearby vegetation, such as leaves; second, the immobilised insect or insect parts were applied to the exposed wound surface using the fingers, often repeatedly, and in some instances temporarily held in the mouth or between the lips before being reapplied; and third, the insect was either discarded and removed from the wound area, with no cases of ingestion recorded. This sequence mirrors that reported in Central chimpanzees, suggesting a potential consistency in techniques across subspecies. Although based on a small sample size, the findings suggest that the behaviour is more widespread than previously assumed^37^, adding to current understanding of behavioural social transmission and prosocial behaviour in chimpanzees. Hence, as a catalyst to guide future research into insect applications, their possible emergence and function, as well as their relevance to the evolution of human medical behaviours, we postulate three different hypotheses focusing specifically on (i) selectivity of the applied insect, (ii) social transmission of insect application, and (iii) other-directed application as a prosocial behaviour.

### Insect selectivity

All observed events involved flying insects rather than other types of insects such as ground-dwelling or crawling insects, consistent with reports from Central chimpanzees^37^. This repeated observation may suggest some degree of selectivity in the types of insects chosen for application. A key question is whether chimpanzees select for these flying insects and a specific taxa for application on their open wounds and whether these applied insects have medicinal value.

In humans, flying insects used for wound healing such as common green bottle flies (*Lucilia sericata*)^45–50^, blow flies (*Sarconesiopsis magellanica*)^51,52^, as well as honeybees (*Apis mellifera*)^53^, and silk moths (*Bombyx mori*)^54,55^, serve several topical functions, including disinfection, antibacterial properties, enhanced healing, tissue reconstruction, and inflammation reduction^3^. Given these established therapeutic properties of various flying insect species in humans, chimpanzees may similarly select flying insects with medicinal properties for wound-related applications. However, chimpanzees may use these insects through mechanisms that also involve systemic effects. *Event 1*, where a white residue was consumed, could indicate systemic benefits alongside topical application. Whether the ingestion serves a function, and what mechanisms (solely topical, or both topical and systemic) might be involved in the insect application, requires further investigation.

Given the difficulty of determining species-specific selection by chimpanzees in real time, future research could address this through a two-fold approach. First, species identification can be achieved through collecting insect remains from application events and identifying them based on morphological characteristics or through molecular techniques such as DNA barcoding^56^. These identifications should be contextualised within the local insect diversity, to enable assessment of whether chimpanzees preferentially select specific species from a wider range of available taxa. Second, assessing whether identified insects have medicinal value and contain compounds relevant to wound healing, such as antibacterial enzymes, anti-inflammatory agents, or other pharmacologically active substances, may be achieved through molecular profiling^57^. Comparing insects used across different chimpanzee populations may offer further insight into whether insect application reflects an adaptive response. If insect application aids wound disinfection, it may provide both therapeutic and prophylactic benefits, with immediate effects (e.g. antimicrobial action) and longer-term benefits (e.g. quicker recovery or reduced infection risk), representing a behaviour with clear fitness advantages. Such information may also provide insight into the potential social transmission of the behaviour – if the same or similar insect species are consistently applied – and into the possibility of other-directed application as a form of prosocial behaviour, particularly if the applied insects have medicinal value (discussed further in the subsequent sections).

### Social transmission of insect application

When delving into the insect application itself and its general sequential pattern, insect applications may necessitate the recognition and handling of specific insect species, which could require social learning to be reliably expressed. One behaviour that may facilitate the transmission of insect application and the sequence is peering^58,59^, as it can provide an opportunity to acquire the knowledge of the behaviour. This is supported by opportunities to observe conspecifics, particularly socially tolerant individuals^60^, who allow close proximity and repeated exposure to their behaviour^61^. Such opportunities are more likely among kin, such as mother-infant pairs, bonded individuals^62^, or role models such as older individuals^60^. In chimpanzees, fission–fusion dynamics lead to changes in group composition and party size^63^, resulting in variation in the availability of role models and a high degree of social tolerance^60^. The acquisition of more complex behaviours, such as insect application, may therefore depend on extended exposure to experienced chimpanzees in tolerant contexts, allowing for more detailed and reliable transmission of information^64^.

In this study, third-party peering was observed during insect application events (Table 1, *N* = 3 of five events), suggesting that these interactions may offer opportunities for the social transmission of the behaviour and its sequence. Previous studies have linked peering to the acquisition of tool-use behaviours, primarily in feeding contexts^65–67^. In the case of insect application, peering may similarly support the transmission of the behaviour or aspects of its sequence, particularly through interactions with socially tolerant individuals or role models. Therefore, we suggest that future research pay particular attention to the presence of peering during insect application events. Studies may examine whether peering predicts subsequent occurrences of insect application (either self-directed or other-directed) by the observing individual, including similar sequences and insect selection. For instance, with *Event 3* which was a follow-up of *Event 2* with Etta-James who peered during the self-directed application of Damien, soon applied an insect to her brother Damien’s wound. Another additional consideration on the insect selection, is if those individuals that peer also applying insects with medicinal value (given that the applied insects possess such properties) or are they replicating the sequence without selecting effective insects, which could lead to different outcomes. Studies should also consider the identity of the model (e.g. kin, older individuals), the tolerance level (duration spent peering during application without a reaction by the model, e.g., moving away or directed aggression), and whether the peering occurred throughout the entire application or partially, as this may influence learning outcomes^68^.

### Insect other-directed application as a form of prosocial behaviour

In humans, empathic responses to another’s pain are suggested to underlie the emergence of prosocial behaviour^69,70^ (see also^71^. These responses are characterised as automatic emotional reactions to the affective state of others, modulated by higher-order cognitive processes^70^. Archaeological evidence suggests that severely injured and disabled Neanderthals survived for extended periods, implying that they received care and support from conspecifics^72^. This kind of other-regarding behaviour (i.e., targeted helping), potentially driven by empathic responses to another’s pain, may have roots in our last common ancestor with chimpanzees. Although some scholars argue that chimpanzees lack human-like empathy^39,73^, other studies suggest that their behaviour reflects empathy-related tendencies, supported by evidence of targeted helping in the wild as a form of epimeletic behaviour^36,74–80^.

The observed events of other-directed insect applications in Eastern and Central chimpanzees^37^, contribute to the ongoing debate on prosocial behaviours in chimpanzees^38,39^. Insect application was observed between related individuals in both chimpanzee subspecies: in Eastern chimpanzees, between siblings; and in Central chimpanzees, between a mother and her son. The remaining two events in Central chimpanzees involved unrelated individuals, suggesting that, while some instances may reflect kin medication^35^, others point toward a broader pattern of social medication^35^ that extends beyond kinship ties, if shown to have a medicinal function. However, in this study, events were recorded ad libitum; thus, instances of other-directed insect application may be underrepresented.

Future research could therefore explore the possibility that other-directed application is a form of prosocial behaviour. Specifically, if the insects identified in other-directed applications possess medicinal properties, such as antimicrobial or anti-inflammatory effects, this behaviour may be adaptive and could therefore qualify as targeted helping. In this context, the application of insects to others may provide evidence for empathy-related capacities in chimpanzees, linked to prosociality. Consequently, insect application and the marked asymmetry between self- and other-directed instances offers an opportunity to explore the cognitive and emotional mechanisms that underlie early forms of empathy and prosocial behaviour.

### Insect application as a medicative behaviour?

Insect application has not yet been classified as medicative according to the self-medication criteria^27,81^. These criteria typically include: (1) recognition of symptoms or injury, (2) use of a substance not part of the regular diet, (3) observable health benefits following use, and (4) evidence that the substance has biological or pharmacological activity^27,81^. These criteria were originally developed for plant use and self-medication. Therefore, we adapted them to help determine whether self-directed and other-directed insect application is a medicative behaviour:

1. Recognition of wounds.
2. The identified insect and its derived material provide little to no nutritional benefit if consumed.
3. Observable wound healing following use.
4. Evidence that the applied insect has biological or pharmacological activity.

Criteria (1), (2), and (4) can be investigated relatively easily through behavioural observation, dietary analysis, and chemical or microbiological testing. In contrast, criterion (3) may pose more challenges, as healing outcomes can be affected by various confounding factors, including the severity and location of the wound, individual variation in immune response, and environmental conditions. Nevertheless, if criteria (1), (2), and (4) are clearly satisfied, this may provide strong evidence in favour for classifying insect application as a medicative behaviour.

## Conclusion

This study presents the first documented evidence of insect application in Eastern chimpanzees, extending a behaviour previously reported only in Central chimpanzees. This finding expands the known repertoire of wound care in chimpanzees and suggests that insect application may be more prevalent than previously recognised. The documentation of both self-directed and other-directed insect applications points to its potential role within the broader medicinal behavioural repertoire of chimpanzees, offers insight into their cognitive complexity, and introduces a crucial candidate behaviour for understanding the evolution of human medical behaviours. More broadly, these findings highlight the importance of preserving wild chimpanzee populations, not only for their continued survival but also for maintaining the behavioural diversity to which insect application may meaningfully contribute^82^.

## Methods

### Study site and subjects

The individuals observed were members of the Ngogo chimpanzee population living in the Kibale National Park in Uganda. At the time of this study, was divided into two distinct communities: Central and Western^83^, and the member size of the population varied between 180 and 220 individuals. Although originally a single community with blurred socio-spatial boundaries between three subgroups (Western, Central, and Eastern), a series of agonistic events led to a permanent split in 2017, resulting in the formation of two distinct communities within the Ngogo population^83^. The study area is approximately 30 km^2^ and consists of mostly mature and regenerating forest transitional between lowland and montane moist evergreen forests, but also smaller areas of swamp forest^84^.

### Behavioural observations

From November 2021 to March 2022, all events involving insect application were collected by K.K. and D.A.F. ad libitum^85^. All occurrences of open wounds were recorded, and if possible, the cause of the wound was documented. Open wounds included punctures, lesions, lacerations, and small cuts. Healed scars and scabs were excluded. Self-directed and other-directed insect applications were recorded digitally using the CyberTracker programme (version 3.514) on a waterproof smartphone. When possible, video footage of the event was recorded using a digital high-definition camera (Sony FDR-AX53 4 K). The ID of the wounded individual and applicator, behavioural sequence of insect application, wound location, and involvement of peering (i.e., close-range and sustained observation of a conspecific’s activities^86^ were noted for each observed event.

### Ethics statement

The research conducted on the chimpanzees was strictly non-invasive and observational, and was classified as a non-animal experiment under Section V, Article 7 of the German Animal Welfare Act (25 May 1998). It was approved by the Ugandan Wildlife Authority (ref: COD/96/05), the Ugandan National Council for Science and Technology (no. NS295ES), the Makerere University Biological Field Station, and the Ngogo Chimpanzee Project. The research adhered to the ASAB Guidelines^87^ and the IUCN Best Practice Guidelines^88^, both of which set internationally recognised standards for ethical, non-invasive behavioural field research on animals, including primates. These standards guided all aspects of fieldwork, including pre-fieldwork quarantine procedures, the use of surgical masks, and maintaining a distance of approximately 10 m from the chimpanzees.

## Data Availability

Data is provided within the manuscript and video footage of reported events is available at the following repository: https://osf.io/aj5v8/?view_only=575674748c254725bf91a9afb2542cdf.
